# Observability Analysis of DVL/PS Aided INS for a Maneuvering AUV

**DOI:** 10.3390/s151026818

**Published:** 2015-10-22

**Authors:** Itzik Klein, Roee Diamant

**Affiliations:** The Acoustic and Navigation Laboratory (ANL), Department of Marine Technology, University of Haifa, Haifa 3498838, Israel; E-Mail: roeed@univ.haifa.ac.il

**Keywords:** AUV navigation, observability Gramian, Inertial navigation systems, AUV maneuvering

## Abstract

Recently, ocean exploration has increased considerably through the use of autonomous underwater vehicles (AUV). A key enabling technology is the precision of the AUV navigation capability. In this paper, we focus on understanding the limitation of the AUV navigation system. That is, what are the observable error-states for different maneuvering types of the AUV? Since analyzing the performance of an underwater navigation system is highly complex, to answer the above question, current approaches use simulations. This, of course, limits the conclusions to the emulated type of vehicle used and to the simulation setup. For this reason, we take a different approach and analyze the system observability for different types of vehicle dynamics by finding the set of observable and unobservable states. To that end, we apply the observability Gramian approach, previously used only for terrestrial applications. We demonstrate our analysis for an underwater inertial navigation system aided by a Doppler velocity logger or by a pressure sensor. The result is a first prediction of the performance of an AUV standing, rotating at a position and turning at a constant speed. Our conclusions of the observable and unobservable navigation error states for different dynamics are supported by extensive numerical simulation.

## 1. Introduction

Recently, the use of Autonomous Underwater Vehicles (AUVs) for ocean applications has increased considerably. These applications include gathering of scientific data, pollution control, climate monitoring, transmission of images from remote places, seafloor mapping, oceanographic surveys and maintenance of off-shore facilities, to name just a few [1,2]. A typical mission of an AUV involves submerging for a few hours while following a specific route and gathering measurements with respect to the AUV’s location and dynamics. To accomplish its mission, the AUV must navigate while being submerged. Initially, the vehicle is assumed to be on the surface and location is determined, commonly, using one of the Global Navigation Satellite Systems (GNSS). However, when the vehicle is below the water surface, GNSS is no longer available and the vehicle must use its inertial system.

Advances made in the last 20 years in Inertial Navigation Systems (INS) technology, in terms of cost, size, and power consumption [3], together with the fact that INS is a standalone system, appoint the INS as the main sensor for AUV navigation. However, the INS navigation solution drifts with time due to error sources within its inertial sensor measurements. A common performance is a navigation error of about 2 km per hour for a navigation grade INS [4]. For that reason, INS is regularly fused with other sensors [5] or data [6] to produce a bounded navigation solution. The INS position solution is commonly aided by GNSS receivers, acoustic positioning [7,8], or imaging/ranging sonar [9]. In sonar imaging/ranging, template matching, for example, is employed where measured data is compared to a bathymetry map [10]. However, in some situations, neither of those position-aiding types is available. For example, when using GNSS receivers, the vehicle is required to surface. In addition, acoustic poisoning requires deploying transponders at known locations, which for most AUV applications is not possible.

When the AUV lacks position updates for some portion of time, it may obtain velocity updates from a Doppler Velocity Logger (DVL) [11,12,13], and an altitude update from a Pressure Sensor (PS) [14,15]. The measured data is introduced to a navigation filter, commonly a Kalman filter, which carries out the process of sensor fusion. The result is a solution for the navigation in terms of an updated parameter state vector. The latter usually includes the position of the AUV, its velocity, and its orientation. However, due to the lack of position updates, the position solution will diverge along with several other navigation states. To compensate for such state divergence, the AUV can maneuver.

It is well known that maneuvering affects the behavior of the navigation system. Among performance, maneuvering also alters the convergence/divergence characteristics of the navigation state vector [16]. While several attempts quantify the navigation performance of an AUV in various types of motion [17,18], an analytical analysis to understand the effect of maneuvering on the navigation capability has not been made. Such analysis is referred to as the “observability analysis”. That is, at a given system state vector and for a given set of measurements, what are the observable navigation parameters? Answering this question is important for understanding the limitations of the vehicle’s navigation system, to set bounds on the navigation capability and performance, and eventually to assist in AUV mission planning.

In general, observability analysis is defined as a process for determining whether the state vector can be inferred. Since there is no formal criterion available to examine the observability of a general nonlinear system, the linear observability theory is usually employed using the corresponding linearized models. In terrestrial navigation, a common technique is to use the observability Gramian [19]. In this sense, a full rank Gramian matrix indicates an observable system, while a rank deficiency indicates that only a sub-space of the state vector is observable. For proper analysis, the question of which states (or some combination of them) are observable and which are not is of great importance. This question may be answered numerically [20] or analytically. Such an analysis has been made for zero update velocity and angular rate updates [21] or body velocity constraint [22] and in the target tracking field for tracking coordinate turn maneuvering targets [23]. Other approaches to investigate observability, in the navigation field, were mostly focused in GPS/INS fusion. Among them, a control-theoretic approach was proposed in [24,25]. In this approach, the linear time-varying system was approximated by a piecewise constant model, and in each constant segment, a simplified null space test was performed to determine the observability properties. A general linear time-varying model was used [26,27] to investigate the observability properties of INS/GPS errors. In [28] the global observability of the nonlinear INS/GPS system with consideration of the lever arm uncertainty was examined. However, no attempt has been made to analyze the system observability for AUVs.

In this paper, we employ the observability Gramian approach and derive analytically the unobservable subspace for an AUV navigation system as a function of the vehicle’s dynamics. Our contribution is two-fold:

1. A general analytical approach to analyze the observability of an underwater navigation system during maneuvering.
2. Deriving conclusions regarding the limitations of the navigation system for an AUV in various dynamic conditions. Specifically, we focus on an INS/DVL and INS/PS navigation system of an AUV standing, rotating at a position and turning at a constant speed.

The analytical results are verified in numerical simulations.

The structure of this paper is organized as follows: Section 2 describes the problem formulation of an aided INS and formalizes the observability Gramian. Section 3 presents the analytical observability analysis. Section 4 shows the numerical results. Finally, Section 5 gives the conclusions.

## 2. Problem Formulation of Aided INS

Denote *H*(t) to be the measurement matrix, and *Φ*(t) to be the state transition matrix, both defined later. To examine the observability of a nonlinear system (such as the INS aiding problems), the observability Gramian:

(1) $$O\left(t_{0},t_{f}\right)=\int_{t_{0}}^{t_{f}}{\text{Φ}}^{T}\left(t,t_{0}\right)H^{T}\left(t\right)H\left(t\right)\text{Φ}\left(t,t_{0}\right)dt$$

is computed [19]. Using [19], the state vector is observable from the measurements iff the observability Gramian is nonsingular. That is, a full rank Gramian matrix indicates a completely observable system, whereas rank deficiency indicates that only a subspace of the state-vector is observable. The unobservable subspace of the state vector can be found by deriving the right null-space of the Gramian matrix such that:

(2) $$H\left(t\right)\text{Φ}\left(t,t_{0}\right)u_{o}=0_{M}$$

where *M* is the number of measurements in each time and *u*_0_ is a set of solutions which span the unobservable subspace of the state vector. To formalize this:

(3) $$null\left[O\left(t_{0},t_{f}\right)\right]=span\left(u_{o}\right)$$

For each type of dynamics examined in this research, our objective is to calculate *u*_0_ from Equation (2) and to multiply it with the state vector in order to find the unobservable subspace, defined as *UOS*.

### 2.1. INS Error State Model

The INS equations of motion, which describes the vehicle position, velocity, and attitude, are nonlinear and thus require a nonlinear filter when fusing INS with additional sensors/information. Commonly, an error-state Extended Kalman Filter (EKF) implementation is used. Let *δx* be the INS error state vector including position error, velocity error, misalignment errors, accelerometer bias residuals and gyro bias residuals, such that $\delta x={\left[\begin{array}{ccccc} \delta p^{n} & \delta v^{n} & \varepsilon^{n} & \delta b_{a} & \delta b_{g} \end{array}\right]}^{T}$, respectively.

The linearized error state differential equations are given in matrix form using [5]:

(4) $$\left[\begin{array}{c} \delta {\dot{p}}^{n}\\ \delta {\dot{v}}^{n}\\ {\dot{\varepsilon }}^{n}\\ \delta {\dot{b}}_{a}\\ \delta {\dot{b}}_{g} \end{array}\right]=\left[\begin{array}{ccccc} 0_{3\times 3} & I_{3} & 0_{3\times 3} & 0_{3\times 3} & 0_{3\times 3}\\ 0_{3\times 3} & 0_{3\times 3} & -\left(f^{n}\times \right) & T^{b\to n} & 0_{3\times 3}\\ 0_{3\times 3} & 0_{3\times 3} & 0_{3\times 3} & 0_{3\times 3} & T^{b\to n}\\ 0_{3\times 3} & 0_{3\times 3} & 0_{3\times 3} & 0_{3\times 3} & 0_{3\times 3}\\ 0_{3\times 3} & 0_{3\times 3} & 0_{3\times 3} & 0_{3\times 3} & 0_{3\times 3} \end{array}\right]\left[\begin{array}{c} \delta p^{n}\\ \delta v^{n}\\ \varepsilon^{n}\\ \delta b_{a}\\ \delta b_{g} \end{array}\right]$$

where $T^{b\to n}$ is the transformation matrix from body to navigation frame and $\left(f^{n}\times \right)$ is the skew symmetric from of the specific force expressed in the navigation frame. The state transition matrix of the system matrix in Equation (4) has a closed form solution:

(5) $$\text{Φ}\left(t,t_{0}\right)=\left[\begin{array}{ccccc} I_{3} & \left(t-t_{0}\right)I_{3} & P_{t} & Q_{t} & T_{t}\\ 0_{3\times 3} & I_{3} & S_{t} & R_{t} & M_{t}\\ 0_{3\times 3} & 0_{3\times 3} & I_{3} & 0_{3\times 3} & R_{t}\\ 0_{3\times 3} & 0_{3\times 3} & 0_{3\times 3} & I_{3} & 0_{3\times 3}\\ 0_{3\times 3} & 0_{3\times 3} & 0_{3\times 3} & 0_{3\times 3} & I_{3} \end{array}\right]$$

where the sub-matrixes are defined as:

(6) $$\begin{array}{ccc} M_{t}=-\int_{t_{0}}^{t}\left[f^{n}\left(s\right)\times \right]R_{s}ds & P_{t}=\int_{t_{0}}^{t}S_{s}ds & R_{t}=\int_{t_{0}}^{t}T_{b}^{n}\left(\tau \right)d\tau \\ S_{t}=-\int_{t_{0}}^{t}\left[f^{n}\left(\tau \right)\times \right]d\tau & Q_{t}=\int_{t_{0}}^{t}R_{s}ds & T_{t}=\int_{t_{0}}^{t}M_{r}dr \end{array}$$

### 2.2. Measurement Models

Two types of aiding sensors are considered herein for INS updates—(1) DVL and (2) PS. These sensors are the usually employed in every AUV. In this section, the corresponding measurement matrixes are described.

#### 2.2.1. DVL

Assuming no lever-arm between the DVL frame and the body frame, only a difference in orientation between the DVL frame and the body frame with transformation matrix $T^{d\to b}$, the DVL measured velocity, ${\tilde{v}}^{d}$, is expressed in the navigation frame as:

(7) $${\tilde{v}}^{n}=T^{b\to n}T^{d\to b}{\tilde{v}}^{d}$$

where $T^{b\to n}$ is the transformation matrix between the body and navigation frames. For simplicity, we assume that $T^{d\to b}$ is accurately known. Thus, Equation (7) becomes:

(8) $${\tilde{v}}^{b}=T^{n\to b}{\tilde{v}}^{n}$$

To obtain the measurement residual we linearize Equation (8):

(9) $$\delta v_{DVL}^{b}=T^{n\to b}\delta v^{n}-T^{n\to b}\left(v^{n}\times \right)\delta \varepsilon$$

where $\delta v_{DVL}^{b}$ is the measurement residual.

The corresponding measurement matrix relating the measurement residual to the error-state is:

(10) $$H_{DVL}=\left[\begin{array}{cccc} 0_{1\times 3} & T^{n\to b} & -T^{n\to b}\left(v^{n}\times \right) & 0_{1\times 6} \end{array}\right]$$

#### 2.2.2. PS

The PS determines the depth of the vehicle by measuring the water pressure. The pressure sensor measurement ${\tilde{h}}_{ps}$ may be modeled as the true depth plus a zero mean Gaussian white noise, *i.e.*:

(11) $${\tilde{h}}_{ps}=h_{true}+w_{ps}$$

Since the altitude is measured directly, the measurement residual is:

(12) $$\delta h^{n}=h_{ins}-{\tilde{h}}_{ps}$$

and the corresponding measurement matrix becomes:

(13) $$H_{ps}=\left[\begin{array}{cc} e_{3} & 0_{1\times 12} \end{array}\right],\;e_{3}=\left[\begin{array}{ccc} 0 & 0 & 1 \end{array}\right]$$

## 3. Analytical Observability Analysis for INS/DVL/PS System

In this section, we present an analytical approach to find the set of unobservable states for AUV navigation using a DVL and a PS. For each case, we give an example of the analysis for the common cases of a stationary vehicle, a turning vehicle, and a vehicle traveling in constant speed. These dynamic types provide an insight towards the vehicle navigation performance while maneuvering.

### 3.1. DVL Assisted Navigation

Since the DVL measures the vehicle velocity, the position error states are unobservable and thus removed from the analysis. The resulting transition matrix and measurement matrix are reduced to:

(14) $$H=\left[\begin{array}{cccc} T_{n}^{b} & -T_{n}^{b}\left(v^{n}\times \right) & 0_{3\times 3} & 0_{3\times 3} \end{array}\right]$$

(15) $${\text{Φ}}_{v}\left(t,t_{0}\right)=\left[\begin{array}{cccc} I_{3} & S_{t} & R_{t} & M_{t}\\ 0_{3\times 3} & I_{3} & 0_{3\times 3} & R_{t}\\ 0_{3\times 3} & 0_{3\times 3} & I_{3} & 0_{3\times 3}\\ 0_{3\times 3} & 0_{3\times 3} & 0_{3\times 3} & I_{3} \end{array}\right]$$

For each maneuvering type, we find the set of unobservable error states by applying the following procedure. Recall *u*_0_ is the set of non-zero solutions of Equation (2), and is the unobservable subspace of the state vector:

(16) $$u_{0}=\left[\begin{array}{cccc} u_{1}^{T} & u_{2}^{T} & u_{3}^{T} & u_{4}^{T} \end{array}\right]$$

Substituting Equations (6) and (14)–(16) into Equation (2) yields:

(17) $$I_{3}u_{1}^{T}+\left[S_{t}-\left(v^{n}\times \right)\right]u_{2}^{T}+R_{t}u_{3}^{T}+\left[M_{t}-\left(v^{n}\times \right)R_{t}\right]u_{4}^{T}=0_{3\times 1}$$

Since the above set of equations hold for any time period, we choose *t* = *0*, and reduce Equation (17) to:

(18) $$u_{1}^{T}=\left(v_{0}^{n}\times \right)u_{2}^{T}$$

Differentiating Equation (18) yields:

(19) $$\left[-\left(f^{n}\times \right)-\left(a^{n}\times \right)\right]u_{2}^{T}+T_{b}^{n}u_{3}^{T}+\left[-\left(f^{n}\times \right)R_{t}-\left(a^{n}\times \right)R_{t}-\left(v^{n}\times \right)T_{b}^{n}\right]u_{4}^{T}=0_{3\times 1}$$

Denote:

(20) $$A^{n}=-\left(f_{0}^{n}\times \right)-\left(a_{0}^{n}\times \right)$$

and define relation:

(21) $${\left(\circ \right)}^{n}=T_{b}^{n}{\left(\circ \right)}^{b}T_{n}^{b}$$

where (∘) is any matrix in ${\text{ℝ}}^{3\times 3}$. Then, substituting Equation (20) into Equation (19) entails:

(22) $$A^{b}T_{n}^{b}u_{2}^{T}+I_{3}u_{3}^{T}+\left[A^{b}T_{n}^{b}R_{t}-\left(v^{b}\times \right)\right]u_{4}^{T}=0_{3\times 1}$$

Differentiating Equation (22) and rearranging gives:

(23) $$\left[{\dot{A}}^{b}T_{n}^{b}-A^{b}{\text{Ω}}_{nb}^{b}T_{n}^{b}\right]u_{2}^{T}+\left[\left({\dot{A}}^{b}T_{n}^{b}-A^{b}{\text{Ω}}_{nb}^{b}T_{n}^{b}\right)R_{t}+A^{b}-\left(a^{b}\times \right)\right]u_{4}^{T}=0_{3\times 1}$$

and differentiating Equation (23) yields:

(24) $$Gu_{2}^{T}+\left[GR_{t}+\left({\dot{A}}^{b}T_{n}^{b}-A^{b}{\text{Ω}}_{nb}^{b}T_{n}^{b}\right)T_{b}^{n}+{\dot{A}}^{b}\right]u_{4}^{T}=0_{3\times 1}$$

where $G=\left[-2{\dot{A}}^{b}{\text{Ω}}_{nb}^{b}T_{n}^{b}+A^{b}{\text{Ω}}_{nb}^{b}{\text{Ω}}_{nb}^{b}T_{n}^{b}\right]$.

Moreover, recall the body acceleration and specific force are given by:

(25) $$\begin{array}{l} a^{b}={\dot{v}}^{b}+\omega_{ib}^{b}\times v^{b}\\ f^{b}=a^{b}-g^{b}=a^{b}-T_{n}^{b}g^{n} \end{array}$$

respectively, where $\omega_{ib}^{b}$ is the measured angular velocity of the vehicle (gyros output) and the measured specific force vector takes into account accelerations due to control, gravity and angular velocity. Using the notation in Equation (25) and assuming zero jerk, *i.e.*, ${\ddot{v}}^{b}=0$ and zero angular acceleration, *i.e.*, ${\dot{\omega }}_{ib}^{b}=0$, ones gets:

(26) $$\begin{array}{l} A^{b}=-2\left[\left({\dot{v}}^{b}+\omega_{ib}^{b}\times v^{b}\right)\times \right]+\left[\left(T_{n}^{b}g^{n}\right)\times \right]\\ {\dot{A}}^{b}=-2\left[\left(\omega_{ib}^{b}\times a^{b}\right)\times \right]-\left[\left({\text{Ω}}_{nb}^{b}T_{n}^{b}g^{n}\right)\times \right] \end{array}$$

Finally, solving Equations (18), (22) and (23) and using Equation (26) for any type of vehicle dynamics, the unobservable subspace of the state vector Equation (16) is obtained.

#### 3.1.1. Stationary Vehicle

In the case of zero velocity, *i.e.*, $\omega_{ib}^{b}=0$, $a^{b}=0$ and $f^{n}=-g$, Equations (18), (22) and (23) reduce into:

(27) $$\begin{array}{l} u_{1}^{T}=0\\ I_{3}u_{3}^{T}=\left[\left(T_{n}^{b}g^{n}\right)\times \right]T_{n}^{b}u_{2}^{T}\\ \left[\left(T_{n}^{b}g^{n}\right)\times \right]u_{4}^{T}=0_{3\times 1} \end{array}$$

Solving Equation (27) for vectors *U* yields the matrix:

(28) $$U=\left[\begin{array}{c} \begin{array}{cccc} 0_{1\times 3} & 0_{1\times 3} & 0_{1\times 3} & \begin{array}{ccc} 0 & 0 & 1 \end{array} \end{array}\\ \begin{array}{cccc} 0_{3\times 3} & I_{3} & -T_{n}^{b}\left(g^{n}\times \right) & 0_{3\times 3} \end{array} \end{array}\right]$$

For simplicity, let us assume the body and navigation frames coincide, *i.e.*, $T_{n}^{b}=I_{3}$. Then, the set of unobservable states is the four dimensional vector:

(29) $$UOS=\left[\begin{array}{c} \delta b_{g,z}\\ \varepsilon_{N}-g\delta b_{a,y}\\ \varepsilon_{E}+g\delta b_{a,x}\\ \varepsilon_{D} \end{array}\right]$$

Notice that, for a stationary vehicle, $\left(v_{0}^{n}\times \right)=0_{3\times 3}$. Then, much like in GNSS velocity update, when $T_{n}^{b}=I_{3}$, the DVL measurement matrix Equation (14) reduces into a linear velocity measurement. The result of a similar analysis will then yield exactly the unobservable subspace in Equation (28) (Ramanandan *et al.*, 2011).

#### 3.1.2. Stationary Vehicle with Angular Velocity

Given a direction vector of angular velocity, $\omega_{ib}^{b}$, Equations (18), (22) and (23) reduce into:

(30) $$\begin{array}{l} I_{3}u_{1}^{T}+R_{t}u_{3}^{T}+M_{t}u_{4}^{T}=0_{3\times 1}\\ \left[\left(T_{n}^{b}g^{n}\right)\times \right]T_{n}^{b}u_{2}^{T}+I_{3}u_{3}^{T}+\left[\left(T_{n}^{b}g^{n}\right)\times \right]T_{n}^{b}R_{t}u_{4}^{T}=0_{3\times 1}\\ B^{b}u_{2}^{T}+B^{b}R_{t}u_{4}^{T}+\left[\left(T_{n}^{b}g^{n}\right)\times \right]u_{4}^{T}=0_{3\times 1}\\ C^{b}u_{2}^{T}+\left[C^{b}R_{t}+B^{b}T_{b}^{n}+{\dot{A}}^{b}\right]u_{4}^{T}=0_{3\times 1} \end{array}$$

where

(31) $$\begin{array}{l} B^{b}=\left[-\left({\text{Ω}}_{nb}^{b}T_{n}^{b}g^{n}\right)\times \right]T_{n}^{b}-\left[\left(T_{n}^{b}g^{n}\right)\times \right]{\text{Ω}}_{nb}^{b}T_{n}^{b}\\ C^{b}=\left[-2{\dot{A}}^{b}{\text{Ω}}_{nb}^{b}T_{n}^{b}+A^{b}{\text{Ω}}_{nb}^{b}{\text{Ω}}_{nb}^{b}T_{n}^{b}\right] \end{array}$$

Without the loss of generality, we examine the case where the angular velocity vector changes only the heading angle, *i.e.*, $\omega_{z}=\dot{\psi }$. In this case, we have:

(32) $$\begin{array}{l} T_{n}^{b}=\left[\begin{array}{ccc} \cos\left(\psi \right) & \sin\left(\psi \right) & 0\\ -\sin\left(\psi \right) & \cos\left(\psi \right) & 0\\ 0 & 0 & 1 \end{array}\right],\;R^{t}=\left[\begin{array}{ccc} \frac{\sin\left(\psi \right)}{\omega_{z}} & \frac{\cos\left(\psi \right)}{\omega_{z}} & 0\\ -\frac{\cos\left(\psi \right)}{\omega_{z}} & \frac{\sin\left(\psi \right)}{\omega_{z}} & 0\\ 0 & 0 & t \end{array}\right]\\ M^{t}=\frac{g}{\omega_{z}^{2}}\left[\begin{array}{ccc} \sin\left(\psi \right) & \cos\left(\psi \right) & 0\\ -\cos\left(\psi \right) & \sin\left(\psi \right) & 0\\ 0 & 0 & 0 \end{array}\right] \end{array}$$

Next, solving the third equation in Equation (30) we get:

(33) $$g\left[\begin{array}{ccc} \cos\left(\psi \right) & \sin\left(\psi \right) & 0\\ -\sin\left(\psi \right) & \cos\left(\psi \right) & 0\\ 0 & 0 & 0 \end{array}\right]u_{2}^{T}=0_{3\times 1}$$

which renders $u_{21}=u_{22}=0$ and $u_{23}=1$. Using the latter result with the last equation of (30) gives:

(34) $$g\omega_{z}\left[\begin{array}{ccc} 1 & 0 & 0\\ 0 & 1 & 0\\ 0 & 0 & 0 \end{array}\right]u_{4}^{T}=0_{3\times 1}$$

which entails $u_{41}=u_{42}=0$. Then, the second equation in Equation (30) yields:

(35) $$g\left[\begin{array}{ccc} \sin\left(\psi \right) & -\cos\left(\psi \right) & 0\\ \cos\left(\psi \right) & \sin\left(\psi \right) & 0\\ 0 & 0 & 0 \end{array}\right]u_{2}^{T}+I_{3}u_{3}^{T}+\frac{g}{\omega_{z}}\left[\begin{array}{ccc} 1 & 0 & 0\\ 0 & 1 & 0\\ 0 & 0 & 0 \end{array}\right]u_{4}^{T}=0_{3\times 1}$$

Substituting Equations (33) and (34) into Equation (35) provides $u_{3}=\left[\begin{array}{ccc} 0 & 0 & 0 \end{array}\right]$. Last, from the first equation in Equation (30) we get $u_{1}=\left[\begin{array}{ccc} 0 & 0 & 0 \end{array}\right]$. The result is the two-dimensional unobservable subspace,

(36) $$UOS=\left[\begin{array}{c} \varepsilon_{D}\\ b_{g,z} \end{array}\right]$$

#### 3.1.3. Vehicle Traveling With Constant Speed and Angular Velocity

For a general direction vector of angular velocity, $\omega_{ib}^{b}$, Equations (18), (22) and (23) reduce into:

(37) $$\begin{array}{l} I_{3}u_{1}^{T}-\left(v^{n}\times \right)u_{2}^{T}+R_{t}u_{3}^{T}+\left[M_{t}-\left(v^{n}\times \right)R_{t}\right]u_{4}^{T}=0_{3\times 1}\\ \left[W^{b}+\left(T_{n}^{b}g^{n}\right)\times \right]T_{n}^{b}u_{2}^{T}+I_{3}u_{3}^{T}+\left[W^{b}+\left(T_{n}^{b}g^{n}\right)\times \right]T_{n}^{b}R_{t}u_{4}^{T}=0_{3\times 1}\\ D^{b}u_{2}^{T}+D^{b}R_{t}u_{4}^{T}+\left[W^{b}+\left(T_{n}^{b}g^{n}\right)\times \right]u_{4}^{T}=0_{3\times 1}\\ C^{b}u_{2}^{T}+\left[C^{b}R_{t}+B^{b}T_{b}^{n}+{\dot{A}}^{b}\right]u_{4}^{T}=0_{3\times 1} \end{array}$$

where

(38) $$\begin{array}{l} D^{b}=\left[-\left({\text{Ω}}_{nb}^{b}T_{n}^{b}g^{n}\right)\times \right]T_{n}^{b}-\left[W^{b}+\left(T_{n}^{b}g^{n}\right)\times \right]{\text{Ω}}_{nb}^{b}T_{n}^{b}\\ W^{b}=-2\left[\left(\omega_{ib}^{b}\times v^{b}\right)\times \right] \end{array}$$

Without the loss of generality, we examine the case where the angular velocity vector changes only the heading angle, *i.e.*, $\omega_{z}=\dot{\psi }$. Then, the velocity vector is $v^{b}={\left[\begin{array}{ccc} v_{x} & 0 & 0 \end{array}\right]}^{T}$. Solving the set of Equation (37) for this case yields the one-dimensional unobservable subspace:

(39) $$UOS=\varepsilon_{D}+2\omega_{z}v_{x}\delta b_{a,x}$$

### 3.2. PS Assisted Navigation

For a PS assisted navigation system, utilizing the same methodology presented above for a INS/DVL system, the unobservable subspace of the state vector, *u*_0_ , is the five dimensional state vector,

(40) $$u_{0}=\left[\begin{array}{cc} \begin{array}{cccc} u_{1}^{T} & u_{2}^{T} & u_{3}^{T} & u_{4}^{T} \end{array} & u_{5}^{T} \end{array}\right]$$

where $u_{i}=\left[\begin{array}{ccc} u_{i1} & u_{i2} & u_{i3} \end{array}\right]$. Substituting Equation (37), the measurement matrix Equation (13), and the transition matrix Equation (4) into Equation (2), one obtains:

(41) $$e_{3}I_{3}u_{1}^{T}+\left(t-t_{0}\right)e_{3}I_{3}u_{2}^{T}+e_{3}P_{t}u_{3}^{T}+e_{3}Q_{t}u_{4}^{T}+e_{3}T_{t}u_{5}^{T}=0$$

Taking the first, second, third and forth derivatives of Equation (41) gives:

(42) $$e_{3}I_{3}u_{2}^{T}+e_{3}S_{t}u_{3}^{T}+e_{3}R_{t}u_{4}^{T}+e_{3}M_{t}u_{5}^{T}=0$$

(43) $$e_{3}\left[-\left(f^{n}\times \right)\right]u_{3}^{T}+e_{3}T_{b}^{n}u_{4}^{T}-e_{3}\left(f^{n}\times \right)R_{t}u_{5}^{T}=0$$

(44) $$e_{3}T_{b}^{n}{\text{Ω}}_{nb}^{b}u_{4}^{T}-e_{3}\left(f^{n}\times \right)T_{b}^{n}u_{5}^{T}=0$$

(45) $$e_{3}T_{b}^{n}{\text{Ω}}_{nb}^{b}{\text{Ω}}_{nb}^{b}u_{4}^{T}-e_{3}\left(f^{n}\times \right)T_{b}^{n}{\text{Ω}}_{nb}^{b}u_{5}^{T}=0$$

from which the set of unobservable states is derived.

#### 3.2.1. Stationary Vehicle

In this case, Equations (42) and (43) reduce into:

(46) $$\begin{array}{l} e_{3}I_{3}u_{1}^{T}=0\Rightarrow u_{13}=0\\ e_{3}I_{3}u_{2}^{T}=0\Rightarrow u_{23}=0\\ e_{3}\left[-\left(f^{n}\times \right)\right]u_{3}^{T}+e_{3}T_{b}^{n}u_{4}^{T}=0 \end{array}$$

Assuming $T_{b}^{n}=I_{3}$, Equation (43) becomes:

(47) $$\begin{array}{l} e_{3}\left[\begin{array}{ccc} 0 & -g & 0\\ g & 0 & 0\\ 0 & 0 & 0 \end{array}\right]u_{3}^{T}+e_{3}T_{b}^{n}u_{4}^{T}=0\\ \Rightarrow u_{43}=0 \end{array}$$

Finally, multiplying the resulted unobservable sub-space by the state vector give the 12 dimensional unobservable subspace,

(48) $$UOS={\left[\delta N\;\delta E\;\delta v_{n}\;\delta v_{e}\;\varepsilon_{N}\;\varepsilon_{E}\;\varepsilon_{D}\;b_{a,x}\;b_{a,y}\;b_{g,x}\;b_{g,y}\;b_{g,z}\right]}^{T}$$

#### 3.2.2. Stationary Vehicle with Angular Velocity

Similar to the case of DVL assisted navigation, we examine the case where the angular velocity vector changes only the heading angle, *i.e.*, when $\omega_{z}=\dot{\psi }$. Since all the matrixes in Equations (42)–(45) are left multiplied by *e*_3_ we are mostly interested in their last row. The results are:

(49) $$\begin{array}{l} e_{3}M_{t}=0_{3\times 1},\;e_{3}S_{t}=0_{3\times 1},e_{3}P_{t}=0_{3\times 1},\;e_{3}T_{t}=0_{3\times 1}\\ e_{3}R_{t}=\left[\begin{array}{ccc} 0 & 0 & t_{0} \end{array}\right],\;e_{3}Q_{t}=\left[\begin{array}{ccc} 0 & 0 & \frac{t_{0}^{2}}{2} \end{array}\right] \end{array}$$

and Equations (42)–(44) reduce into:

(50) $$\begin{array}{l} u_{13}+t_{0}^{2}/2u_{43}=0\\ u_{23}+t_{0}u_{43}=0\\ u_{43}=0 \end{array}$$

Solving Equation (50) for vector u yields $u_{13}=u_{23}=u_{43}=0$. This is the same result as without the rotation. Thus, the unobservable subspace is spanned by Equation (48). However, since all the matrixes in Equations (42)–(45) are left multiplied by *e*_3_, the direction of the angular velocity vector will ultimately affect differently the unobservable subspace. To demonstrate that, let the angular velocity vector change only the pitch angle, *i.e.*, $\omega_{y}=\dot{\theta }$. In this case,

(51) $$\begin{array}{l} A^{n}=-\left(f_{0}^{n}\times \right)-\left(a_{0}^{n}\times \right)T_{n}^{b}=\left[\begin{array}{ccc} \cos\left(\theta \right) & 0 & -\sin\left(\theta \right)\\ 0 & 1 & 0\\ \sin\left(\theta \right) & 0 & \cos\left(\theta \right) \end{array}\right]\\ {R^{t}|}_{t=t_{0}}=\frac{1}{\omega_{y}}\left[\begin{array}{ccc} \sin\left(\theta \right) & 0 & \cos\left(\theta \right)\\ 0 & \omega_{y}t_{0} & 0\\ -\cos\left(\theta \right) & 0 & \sin\left(\theta \right) \end{array}\right],{T^{t}|}_{t=t_{0}}=\left[\begin{array}{ccc} {}* & * & *\\ {}* & * & *\\ 0 & 0 & 0 \end{array}\right]\\ {M^{t}|}_{t=t_{0}}=\left[\begin{array}{ccc} {}* & * & *\\ {}* & * & *\\ 0 & 0 & 0 \end{array}\right],{Q^{t}|}_{t=t_{0}}=\frac{1}{\omega_{y}^{2}}\left[\begin{array}{ccc} {}* & * & *\\ {}* & * & *\\ -\sin\left(\theta \right) & 0 & -\cos\left(\theta \right) \end{array}\right]\\ {S^{t}|}_{t=t_{0}}={P^{t}|}_{t=t_{0}}=0_{3\times 3} \end{array}$$

Note that since the matrixes are left multiplied by $\omega_{z}=\dot{\psi }$ $e_{3}$, the elements marked by * are not important.

Substituting Equation (51) into Equations (42)–(45) yields:

(52) $$\begin{array}{l} u_{13}-\frac{\sin\left(\theta \right)}{\omega_{y}^{2}}u_{41}-\frac{\cos\left(\theta \right)}{\omega_{y}^{2}}u_{43}=0\\ u_{23}-\frac{\cos\left(\theta \right)}{\omega_{y}}u_{41}+\frac{\sin\left(\theta \right)}{\omega_{y}}u_{43}=0\\ \sin\left(\theta \right)u_{41}+\cos\left(\theta \right)u_{43}=0\\ u_{41}=0 \end{array}$$

Solving Equation (52) for vector u yields $u_{13}=u_{23}=u_{43}=u_{41}=0$. Here, the rotation allows also the estimation of the x-accelerometer bias. The results is the 11 dimensional unobservable subspace:

(53) $$UOS={\left[\delta N\;\delta E\;\delta v_{n}\;\delta v_{e}\;\varepsilon_{N}\;\varepsilon_{E}\;\varepsilon_{D}\;b_{a,y}\;b_{g,x}\;b_{g,y}\;b_{g,z}\right]}^{T}$$

#### 3.2.3. Vehicle Traveling with Constant Speed and Angular Velocity

For this considered case,

(54) $$\begin{array}{l} {R^{t}|}_{t=t_{0}}=\left[\begin{array}{ccc} {}* & * & *\\ {}* & * & *\\ 0 & 0 & t_{0} \end{array}\right],{M^{t}|}_{t=t_{0}}=\left[\begin{array}{ccc} {}* & * & *\\ {}* & * & *\\ 0 & \left(v_{x}/\omega_{z}\right)t_{0} & 0 \end{array}\right]\\ ,{T^{t}|}_{t=t_{0}}=\left[\begin{array}{ccc} {}* & * & *\\ {}* & * & *\\ 0 & \frac{v_{x}t_{0}^{2}}{2\omega_{z}} & 0 \end{array}\right],{Q^{t}|}_{t=t_{0}}=\left[\begin{array}{ccc} {}* & * & *\\ {}* & * & *\\ 0 & 0 & t_{0}^{2}/2 \end{array}\right]\\ {S^{t}|}_{t=t_{0}}={P^{t}|}_{t=t_{0}}=0_{3\times 3} \end{array}$$

Substituting Equation (54) into Equations (42)–(45) yields:

(55) $$\begin{array}{l} u_{51}=0\\ u_{52}=0\\ u_{13}+\frac{t_{0}^{2}}{2}u_{43}+\frac{v_{x}t_{0}^{2}}{2\omega_{z}}u_{52}=0\\ u_{23}+t_{0}u_{43}+\frac{v_{x}t_{0}}{\omega_{z}}u_{52}=0\\ \omega_{z}v_{x}\cos\left(\psi \right)u_{31}+\omega_{z}v_{x}\sin\left(\psi \right)u_{32}+u_{43}+v_{x}u_{52}=0 \end{array}$$

Then, solving Equation (55) yields the nine-dimensional unobservable subspace:

(56) $$UOS={\left[\delta N\;\delta E\;\delta v_{n}\;\delta v_{e}\;\varepsilon_{E}-\tan\left(\psi \right)\varepsilon_{N}\;\varepsilon_{D}\;b_{a,x}\;b_{g,y}\;b_{g,z}\right]}^{T}$$

## 4. Numerical Analysis

To verify the analytical expressions derived in Section 3, we carried out numerical simulation to emulate the fusion of the INS navigation with information from DVL and from PS. For data fusion we used an error-state EKF with the linearized error state differential Equation (4). Throughout all simulation runs, the initial accelerometers bias standard deviation was 3 mg and the initial gyros bias standard deviation was 3°/h. The results are measured in terms of the rank of the observability Gramian as a function of time for various vehicle dynamics. This rank was compared with the non-observable subspace analyzed in the previous section. A match is found in case the subtraction outcome between the Gramian rank and the system rank is equal to the dimension of the unobservable subspace. To observe which error states divergence and which convergence for each type of dynamics, we also present the estimated EKF standard deviation of the error states.

### 4.1. Simulation Results

#### 4.1.1. INS/DVL Fusion

**Figure 1:**
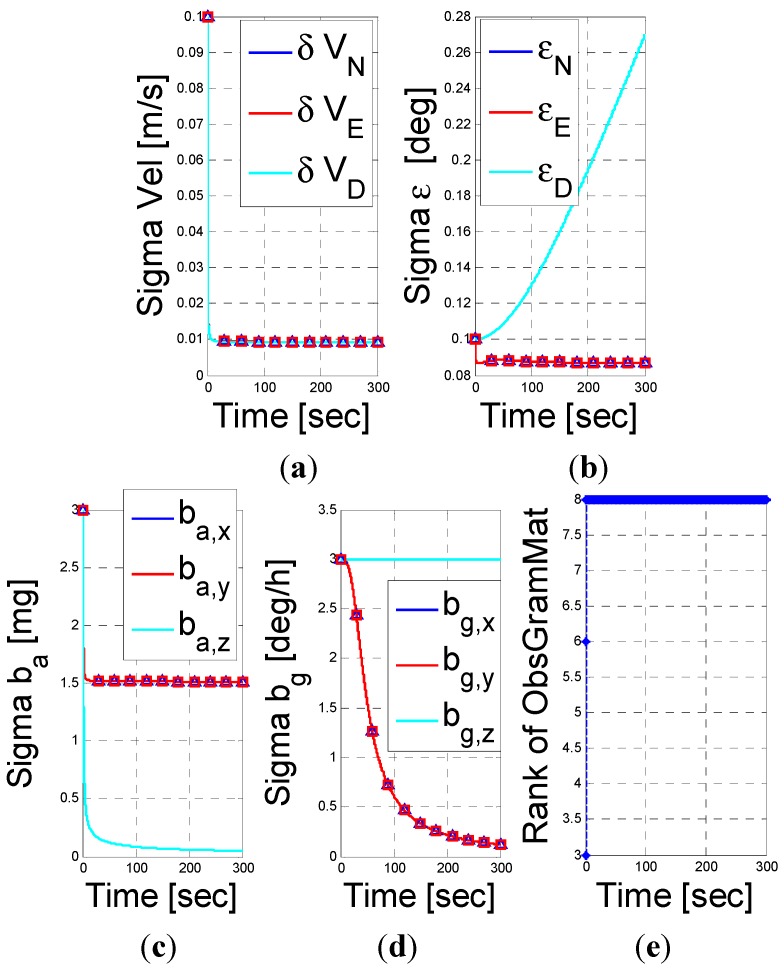
INS/DVL fusion results for a stationery vehicle scenario. (**a**) velocity error vector; (**b**) misalignment error; (**c**) accelerometer residuals; (**d**) gyro residuals; (**e**) rank of observbility Gramian matrix.

First, in Figure 1, we examined the case of a DVL assisted INS navigation with a stationary vehicle. As mentioned earlier, since the DVL measures the vehicle velocity the position error states are not observable and thus removed of the analysis. Consequently, the system dimension is 12 instead of 15. By Equation (29), the UOS vector is four dimensional and thus we expect the rank of the Gramian matrix to be 8. Observing Figure 1, we immediately see that the rank of the Gramian matrix is 8. Additionally, as indicated by the UOS Equation (29), while the other error parameters converge, the down misalignment angle, z-axis gyro bias, x-y axes accelerometer biases standard deviation does not converge.

**Figure 2:**
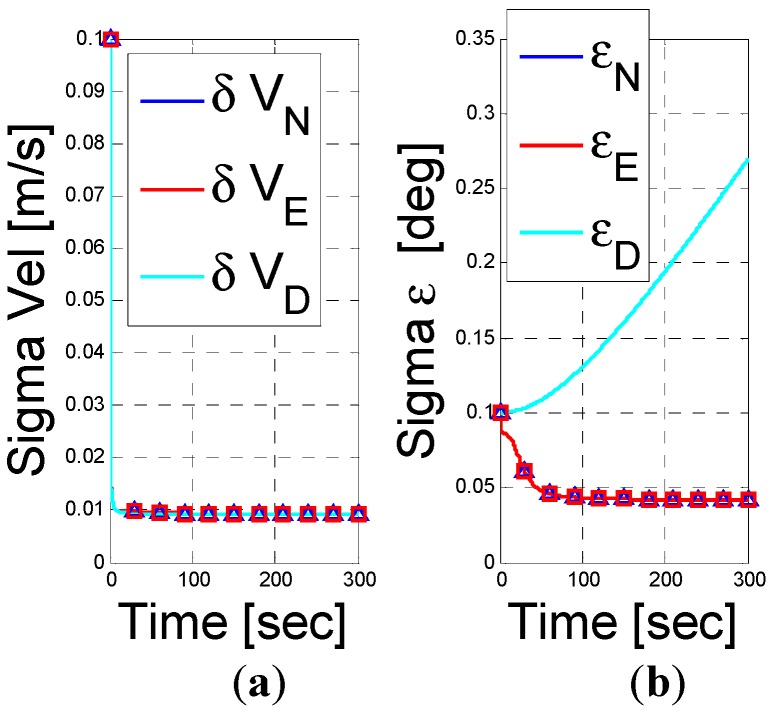

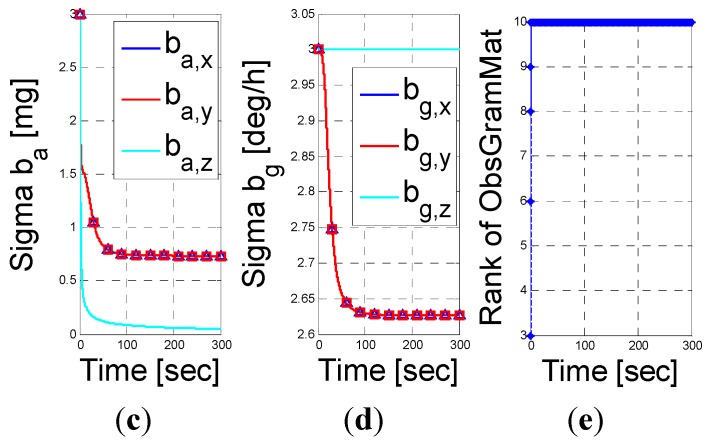
INS/DVL fusion results for a rotating vehicle scenario. (**a**) velocity error vector; (**b**) misalignment error; (**c**) accelerometer residuals; (**d**) gyro residuals; (**e**) rank of observbility Gramian matrix.

Next, we allow the vehicle to rotate around the z-body (1°/s). The results are shown in Figure 2. As analyzed in Equation (36), the unobservable subspace reduces into a two dimensional vector. As foreseen by our analysis, we observe that only the down misalignment angle and z-axis gyro bias are unobservable. We therefore conclude that the rotation helps to observe the x-y axes accelerometer biases and, as illustrated in Figure 2, the rank of the observability Gramian matrix increases to 10.

**Figure 3:**
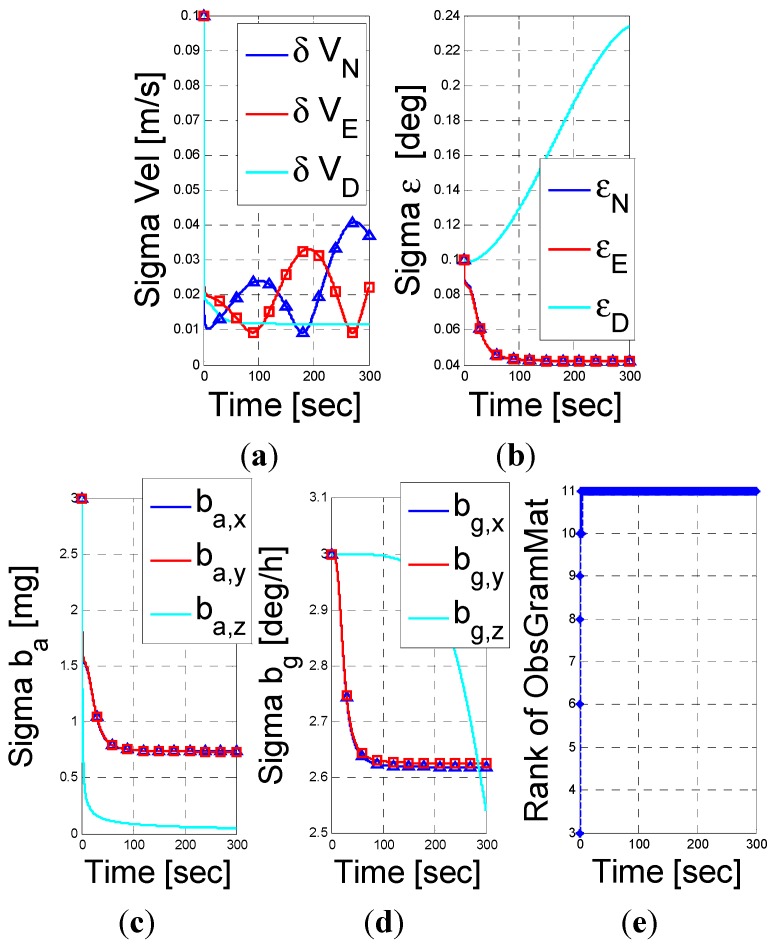
INS/DVL fusion results for a vehicle traveling with constant speed and angular velocity scenario. (**a**) velocity error vector; (**b**) misalignment error; (**c**) accelerometer residuals; (**d**) gyro residuals; (**e**) rank of observbility Gramian matrix.

The case of a vehicle travelling with constant speed (10 m/s) and experiences rotation around the z-body (1°/s) is simulated in Figure 3. As given by Equation (39), the unobservable subspace is shown to be of one-dimensional space, where only a linear combination of the down misalignment angle and z-axis gyro bias is unobservable.

#### 4.1.2. INS/PS Fusion

**Figure 4:**
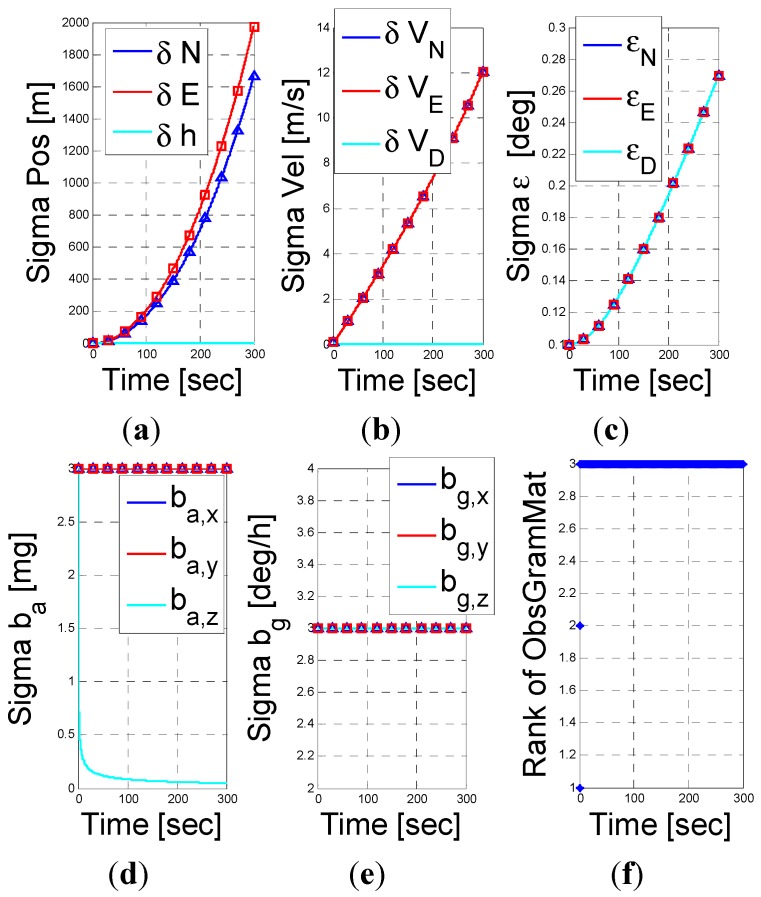
INS/PS fusion results for a stationery vehicle scenario. (**a**) position error vector; (**b**) velocity error vector; (**c**) misalignment error; (**d**) accelerometer residuals; (**e**) gyro residuals; (**f**) rank of observbility Gramian matrix.

Next, we examine the fusion of the PS assisted INS navigation when the vehicle is stationary. According to the result in Equation (48), only the altitude error, down velocity error, and the z-axis accelerometer bias are observable. The numerical results shown in Figure 4 support this conclusion. In addition, as shown in Figure 4, the rank of the observability Garmin is 3, *i.e.*, an unobservable subspace of rank 12. This result supports our analysis.

**Figure 5:**
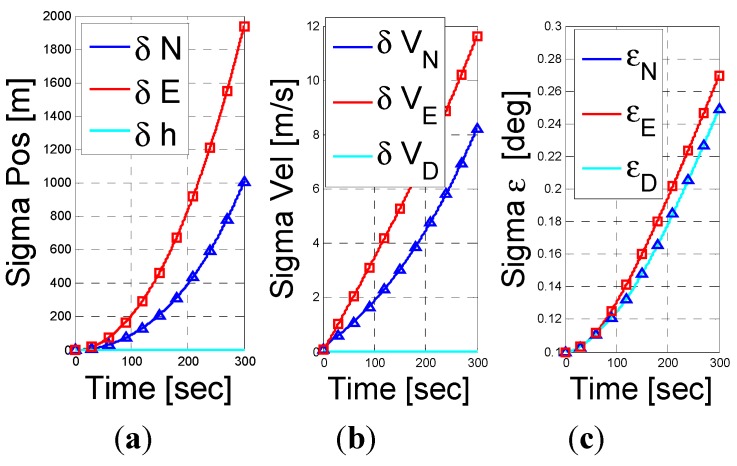

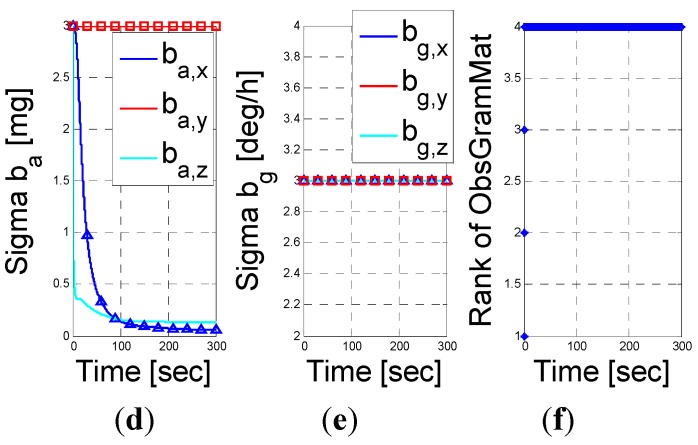
INS/PS fusion results for a rotating vehicle scenario. (**a**) position error vector; (**b**) velocity error vector; (**c**) misalignment error; (**d**) accelerometer residuals; (**e**) gyro residuals; (**f**) rank of observbility Gramian matrix.

When the vehicle also rotates around the y-body at 1°/s then, by Equation (53), the unobservable subspace reduces into a vector of 11 dimensions. That is, the rotation allows also the estimation of the x-accelerometer bias. This is shown in Figure 5.

**Figure 6:**
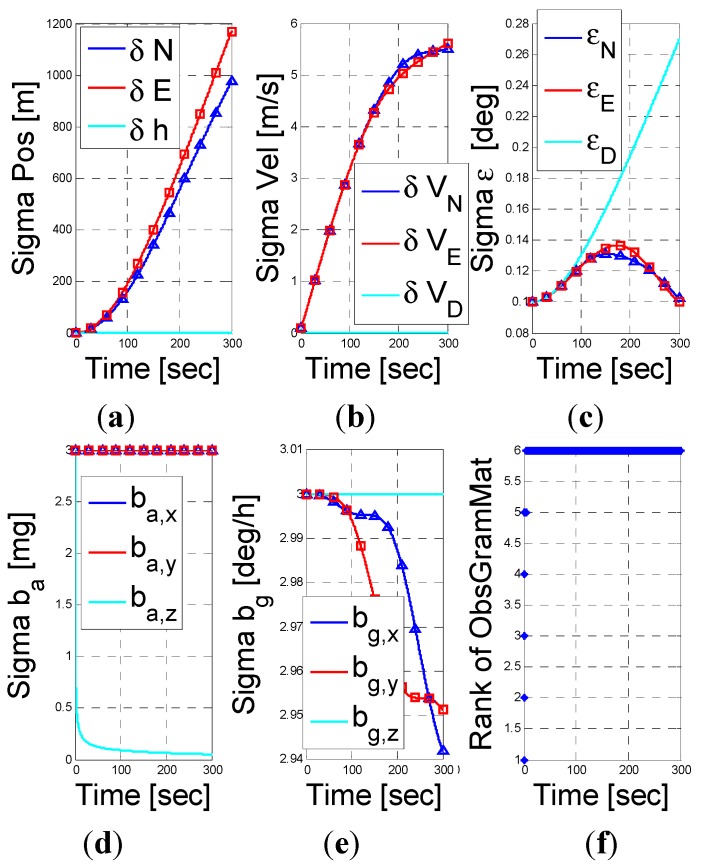
INS/PS fusion results for a vehicle traveling with constant speed and angular velocity scenario. (**a**) position error vector; (**b**) velocity error vector; (**c**) misalignment error; (**d**) accelerometer residuals; (**e**) gyro residuals; (**f**) rank of observbility Gramian matrix.

Finally, for the case of a vehicle travelling with constant speed (10 m/s) and experiences rotation around the z-body (1°/s), we predicted in Equation (48) that the unobservable subspace would be a 9 dimensional one. Recall that by Equation (48), without the constant speed the unobservable subspace is 12 dimensional unobservable subspace. That is, the addition of constant speed to the rotation helps reducing the unobservable subspace. As shown in Figure 6, the addition observable states are the x-y axes gyro bias, and the linear combination of the east and north misalignment turned.

### 4.2. Discussion

Our analysis and simulation for the INS/DVL and INS/PS AUV navigation system showed some interesting results. First, as expected, the observable sub-space becomes larger as maneuvering becomes more complex. That is, perhaps contrary to intuition, AUV path planning should include as much as possible rotating, turning, and motion. In this context, the effect of motion on reducing the unobservable sub-space is the largest. Interestingly, this conclusion applies also for the INS/PS system, which does not include direct measurement of speed.

Second, comparing the results for the INS/DVL and the INS/PS systems, we observe that the observable space for the INS/DVL system has considerably higher rank than that of the INS/PS system. This is an alternative method to observe that a DVL delivers much more information than a PS. Finally, comparing the two navigation systems, we conclude that the effect of maneuvering is considerably higher on the INS/DVL system.

## 5. Conclusions

In this paper, we focused on the problem of predicting the observable and unobservable error states of the AUV navigation system for several maneuvering types. This is important for understanding the limits of the INS AUV navigation system, as well as to improve path planning for AUVs. Our analysis was based on the observability Gramian approach previously used only for terrestrial applications. We demonstrated our analysis for an INS navigation system assisted by a DVL and by a PS. For each of these systems we analyzed and concluded what would be the unobservable subspace of the navigation error state for different dynamic types. Specifically, we considered the three basic maneuvering types an AUV performs, namely, (1) stationary vehicle (2) stationary vehicle with angular rotation and (3) a vehicle traveling with constant speed and angular rotation. Our results clearly show that the addition of rotation and further linear velocity helps reducing the unobservable subspace, *i.e.*, more error states may be estimated from the measurements. The analytical expressions where verified by numerical simulation. Excact match of the analysis was obtained. That is, the number and identity of the observable and unobservable error states obtained in the simulations match with the analysis for the observability Gramian. Future work will include an analytical observability analysis of the INS/DVL/PS system.

## Nomenclature

AUV: Autonomous Underwater Vehicles
DVL: Doppler Velocity Logger
GNSS: Global Navigation Satellite Systems
INS: Inertial Navigation Systems
PS: Pressure Sensor
UOS: Unobservable subspace
*Φ*(t): State transition matrix
*δb_a_*: Accelerometer bias residuals
*δb_g_*: Gyro bias residuals
*δp*: Position error vector
*δv*: Velocity error vector
*δv*_DVL_: DVL velocity measurement residual
*δ**x*: INS error state vector
*ε*: Misalignment errors
*ψ*: Heading angle
*θ*: Pitch angle
*ω*: Angular velocity vector
*H*(t): Measurement matrix
*M*: Number of measurements
*T^b→n^*: Transformation matrix from body to navigation frame
*T^d→b^*: Transformation matrix from DVL to body frame
*a*: Acceleration vector
*f*: Specific force vector
*g*: Gravity vector
*h*: Vehicle depth
*u*_0_: set of solutions which span the unobservable subspace of the state vector
*v*: Velocity vector
(•)*^n^*: A vector expressed in the navigation frame
(•)*^b^*: A vector expressed in the body frame
(•)*^d^*: A vector expressed in the DVL frame
(•)*^i^*: A vector expressed in the inertial frame
